# Mental Health Trajectories of Men and Women Who Start Providing Personal Care: European Findings From SHARE Using Propensity Score Matching

**DOI:** 10.1093/geronb/gbaf053

**Published:** 2025-03-13

**Authors:** Morten Wahrendorf, Anne McMunn, Baowen Xue, Valerie Schaps, Christian Deindl, Giorgio Di Gessa, Rebecca E Lacey

**Affiliations:** Institute of Medical Sociology, Medical Faculty and University Hospital Düsseldorf, Heinrich Heine University Düsseldorf, Düsseldorf, Germany; Research Department of Epidemiology and Public Health, University College London, London, UK; Research Department of Epidemiology and Public Health, University College London, London, UK; Institute of Medical Sociology, Medical Faculty and University Hospital Düsseldorf, Heinrich Heine University Düsseldorf, Düsseldorf, Germany; Department of Social Sciences, TU Dortmund University, Dortmund, Germany; Research Department of Epidemiology and Public Health, University College London, London, UK; School of Health and Medical Sciences, City St George’s, University of London, London, UK; (Social Sciences Section)

**Keywords:** Caring, Depressive symptoms, Europe, Long-term care policies

## Abstract

**Objectives:**

We examine the mental health trajectories of people who start providing personal care and compare their trajectories with matched controls who remain non-carers. We also investigate whether trajectories vary by gender, financial resources, and supportive long-term care policies.

**Methods:**

Using 9 waves of the Survey of Health, Ageing, and Retirement in Europe, collected in 28 European countries from 2004 to 2022, we analyze longitudinal data from 68,075 men and women aged 50 or older. We identify transitions into regular personal care within the household and use depressive symptoms from up to 4 waves before and after transitioning into care to measure mental health trajectories. Financial resources are measured by household wealth, whereas 3 macro indicators assess (1) support for caregivers, (2) support for care recipients, and (3) public care service availability. Propensity score matching, applied separately for men and women, identifies matched noncaregivers from the same country, and we use piecewise growth curve models to examine changes before, during, and after becoming a carer.

**Results:**

Both men and women have a clear increase in depressive symptoms when becoming a regular carer, and this increase even begins before the transition. The increase during the transition is slightly more pronounced for women and those with lower wealth, but we find no systematic differences by policy indicators.

**Discussion:**

Our study highlights the need for improved support for carers. Although national policies may influence the likelihood of becoming a carer, their effectiveness in mitigating the mental health impact of caring remains unclear.

Across Europe, millions of older men and women provide personal care to support ill and disabled adults. In most cases the care is not provided by professional healthcare providers—but rather through family members who are usually unpaid. Depending on the source, studies estimate that up to 75%–90% of all care in Europe is provided in this way (UNECE, 2019). As an indispensable part of current care provision, starting to provide personal care is therefore increasingly likely. It is, therefore, rather surprising that the evidence on how becoming a carer is related to changes in mental health is still restricted, and that only a few longitudinal studies have investigated trajectories of mental health over an extended time period, covering periods before, during, and after the transition into personal care.

In fact, the vast majority of studies in the field are based on cross-sectional studies comparing groups of carers with non-carers (for systematic reviews, see Lacey et al., 2022; Pinquart & Sörensen, 2003). Overall, these studies suggest that personal care (especially intensive personal care that is provided within the household) is associated with poorer mental health. The main explanations given in the literature are the increased psychosocial and physical strain of care (Pearlin et al., 1990), especially in terms of limited reward and restricted control and autonomy (Haidt & Rodin, 1999; McMunn et al., 2009; Wahrendorf et al., 2008), or the inherent sadness, burdens, and worries of having a relative in poor health (Hansen & Slagsvold, 2013; Litwin et al., 2014). However, cross-sectional studies still provide limited evidence on how care is related to mental health. For example, it is not known whether people who provide care already had poorer health before they became carers. This includes potential risks of reverse causality or selection into care of people in poor health. With the growing number of longitudinal studies, these shortcomings are partly addressed, mainly by studies that investigate intraindividual changes in mental health related to changes in caring status based on fixed-effects models (Kaschowitz & Brandt, 2017; Uccheddu et al., 2019) or studies that investigate how mental health changes depend on caring status at a given moment. These studies suggest that mental health declines if people become personal carers, or that caring is linked with worsening health. However, the periods before and after the transition to care are still not really addressed in these studies. For example, it is not clear whether and for how long health differences between carers and non-carers persist after becoming a carer, or whether their health even starts to worsen before they become carers. One notable exception is a recent study by Lacey et al. (2024) based on longitudinal data from the UK. The study uses annual health information for up to 8 years before and 8 years after becoming a carer and compares health information of carers to mental health trajectories of matched non-carers (based on propensity score matching). The findings suggest that mental health worsens during the transition into care and that the mental health difference between carers and non-carers persists for about 3 years after becoming a carer. In addition, they find that sometimes mental health even worsens before people become a carer. This latter finding is consistent with the idea that people often identify themselves as carers rather late in the process, sometimes after realizing that their caring activity goes beyond what is usually considered to be typical family responsibilities in their country context (Montgomery et al., 2007).

Besides the limited longitudinal evidence, mental health trajectories of people who become a carer possibly differ by various factors, including gender, financial resources, and national care policies (Verbakel, 2014). These factors have largely been overlooked in previous research. They may not only affect the likelihood that a person becomes a carer but also moderate the association between starting to provide personal care and mental health. It is well-known, for example, that older women more often provide personal care than men (Eurocare, 2024; McMunn et al., 2020; Pinquart & Sorensen, 2006; Tur-Sinai et al., 2020). It is, however, still not clear if women suffer more from caring than men in older ages, with findings pointing in both directions (Dunkle et al., 2014; Hajek & König, 2016). On the one hand, as care tasks are often more demanding and intense for women than for men (Eurocare, 2024), we may expect that women are more likely to suffer than men. On the other hand, because of cultural preferences and traditional gender roles, one may also assume that men doing intensive care may be more financially deprived (Hajek & König, 2016). Likewise, studies suggest that economically disadvantaged population groups are more likely to provide care, especially intense care within the household (Quashie et al., 2022), and this could lead to stronger detrimental impacts on health. Financial resources may also affect the extent to which carers receive external support, and therefore, reduce the health burden for people with higher incomes. A comprehensive assessment of whether financial resources moderate the association between caring and mental health, however, is still missing.

This directly links to existing country-specific long-term care policies (LTC policies) and the extent to which carers or care recipients (or both) are supported through these policies. For example, one could assume that policies that generously support personal care (e.g., through direct cash benefits) reduce carers’ burden, because it is compensated or as they are more likely to afford professional assistance. As with the aforementioned factors, however, studies to date have mostly investigated if LTC policies increase or decrease the likelihood that people provide care (suggesting that low spending is linked with more frequent intensive caring in a country; Quashie et al., 2022; Verbakel, 2018), rather than their impact on health effects (i.e., moderation). There is some evidence that generous and supportive policies (especially the availability of formal long-term resources) also mean that the negative effects on health are less pronounced (Dujardin et al., 2011; Uccheddu et al., 2019; Verbakel, 2014), but some studies also find that associations were similar across countries (Kaschowitz & Brandt, 2017). To some extent these mixed findings may also be due to unsystematic country comparisons that often did not rely on conceptually based policy measures because comparisons rely on typologies of care regimes that often combine different dimensions of care policies. Against this background, Verbakel and colleagues (2023) have recently suggested three macrolevel indicators of supportive LTC policies to facilitate country comparisons, namely, policies that support persons in charge of care (mostly family members), policies that support care recipients, and policies or measures that provide an infrastructure or services that facilitate professional care outside of the family. In doing so, LTC policies are often also distinguished by the extent that they either reduce or enhance reliance on family support, that is, the “familializing” or “defamilialization” effects (Bambra, 2007a; Leitner, 2003). Policies that support carers, for example, may reinforce the role of the family in a country. In contrast, policies that provide financial resources for persons in need of care or that provide an infrastructure or services for professional care (e.g., public provision of care) are rather supposed to diminish the role of the family in a country (for a detailed discussion, see Bambra, 2007a; Verbakel et al., 2023). As in the case of financial resources, we may assume that all these three types of supportive policies could attenuate the health effects of caring, as they provide alternatives for family-based care or compensation for family carers.

This paper examines the mental health trajectories of older men and women who become carers, comparing them to matched non-carers with similar characteristics. It also investigates differences in trajectories based on gender, wealth, and levels of supportive LTC policies.

## Method

### Data Source

Data come from nine waves of the Survey of Health, Ageing and Retirement in Europe (SHARE, Release 9.0.0), collected between 2004 and 2022. SHARE is a cross-national, longitudinal study that collects sociological, economic, and health-related information at 2-year intervals among older people across Europe as part of an open cohort study (allowing new members/countries to join over time). In each country, participants are selected through probability household sampling, interviewing people aged 50 years or older (plus their partner) using Computer Assisted Personal Interviews (CAPI). First wave data were collected between 2004 and 2006 (in 12 countries including Israel), as well as SHARE also provides life history data (but no information on current circumstances) that is either collected in wave 3 or wave 7 (named “SHARELIFE”). Since the study’s onset, 17 new countries have joined SHARE, as well as new participants were included in countries in the course of the survey to increase sample size and to maintain population representation. The latest ninth wave was between 2021 and 2022. In sum, this results in data for 158,764 respondents who participated at least once. For a detailed data resource profile of SHARE, see elsewhere (Borsch-Supan et al., 2013).

### Study Population

We applied several data restrictions for this study. Participants outside the age range of 50 to 90 years at baseline (when entering the study) were excluded (6,452 out of 158,764 respondents). Individuals living alone were also excluded (29,392 respondents) because caregiving questions were only asked to multipersons households. To capture transitions into care, we included only participants with data on care for at least 2 waves (excluding 47,470 respondents with only one wave of data) and those who were carers at baseline (excluding 5,394 respondents). Next, we excluded participants without information on mental health (additionally excluding 291 out of 70,056), and those with missing data on variables used for the propensity score matching (1,690 participants). This results in a raw study sample of 68,075 respondents from 28 countries (see Table 2 for a list of all countries), varying in number of waves (ranging from 2 to 9 waves, with an average of 4.2 waves) and patterns they participated (depending on when entering the study). Among these, 5,201 women (15.0% of all women) and 3,847 men (11.5% of all men) became carers during the study and are used as treatment cases in the subsequent analyses. Details are provided in a supplementary flowchart (Supplementary Figure S1).

**Table 2.** Policy Indicators by Country

Country	Caregiver support (index)^a^	Cash benefit to care recipient (index)^b^	LTC beds (per 1,000 65+ population)^c^
Austria	**0.86**	**4**	43.5
Germany	**0.86**	**4**	**54.0**
Sweden	**0.71**	0	**70.8**
Netherlands	**0.86**	—	**86.5**
Spain	**0.71**	**4**	45.5
Italy	0.57	**4**	18.3
France	0.57	—	**55.4**
Denmark	**1.00**	0	50.3
Greece	0.57	0	1.9
Switzerland	—	—	—
Belgium	0.43	3	**71.2**
Israel	—	—	—
Czech Republic	0.43	**4**	38.8
Poland	0.00	2	12.5
Luxembourg	**0.86**	3	**56.7**
Hungary	0.57	0	49.2
Portugal	0.43	3	—
Slovenia	0.14	—	**58.4**
Estonia	—	0	41.5
Croatia	—	—	—
Lithuania	0.57	—	34.6
Bulgaria	0.57	3	2.7
Cyprus	—	—	—
Finland	**1.00**	**4**	**61.1**
Latvia	0.57	—	20.1
Malta	0.57	0	**63.2**
Romania	0.14	—	8.4
Slovakia	0.29	—	**51.1**

*Note*s: LTC = long-term care. All values are taken from Verbakel et al. (2023). For subsequent analyses, values in bold are considered as countries with extended policies (labeled as “high” vs “low”).

^a^Values refer to 2012.

^b^Values refer to 2009.

^c^All values refer to 2012 (except Denmark 2011).

### Variables

#### Personal care

As part of the SHARE questionnaire, respondents were asked at each wave (except in SHARELIFE) whether they had provided personal care to a household member on a regular basis in the past 12 months. Specifically, respondents were asked, “Is there someone living in this household whom you have helped regularly during the last twelve months with personal care, such as washing, getting out of bed, or dressing?” To avoid measuring care during short-time sickness, the term “regularly” was clarified as daily or almost daily. Based on this, we created a binary indicator (yes/no) for each wave when the respondent participated and measured whether and when the respondent first began providing regular personal care during the observation period, regardless of later caring patterns.

#### Mental health

Mental health was assessed using the EURO-D depression scale, which includes 12 items measuring the presence (based on binary indicators) of the following depressive symptoms over the past month: “depressed mood,” “pessimism,” “suicidality,” “guilt,” “sleep quality,” “interest,” “irritability,” “appetite,” “fatigue,” “concentration,” “enjoyment,” and “tearfulness.” When summing up the number of symptoms, the scale ranges from 0 to 12, with higher values indicating poorer mental health.

### Wealth

Wealth is taken from baseline and is based on household total net worth, including financial wealth (savings, net stock value, mutual funds, and bonds) and housing wealth (value of primary residence, other real estates, own business share, and cars). We calculated country-specific tertiles based on the raw study sample (low, medium, high). Because wealth reflects accumulated savings rather than household income, it may be better suited for older populations as an indicator of financial resources.

#### Policy indicators

We use three policy indicators described by Verbakel et al. (2023), representing distinct dimensions of supportive LTC policies.

- Caregiver support: This index captures various LTC policies in a country aimed to support care provided through and within the family, also termed “supported familialism.” It includes seven types of carer support (i.e., counseling, information, respite care, training, financial allowance, pension credits, and care leave; see also Courtin et al., 2014). Scores range from 0 to 1 and are provided for 2012 (Courtin et al., 2014; Verbakel et al., 2023), with higher values representing stronger support.
- Cash benefit to care recipient: This index measures financial support provided to care recipients, enabling them to purchase care services. By providing financial support to the care recipient it reflects “defamilialization through the market” (Verbakel et al., 2023), as it enables those in need of care to buy care services outside of the family on the market. In addition to information on the availability of cash benefits in a country, it also uses information on formal regulations (i.e., eligibility criteria) and the extent of support, leading to an index that ranges from 0 (no cash benefits) to 4 (most generous cash benefits). From Verbakel et al. (2023), this indicator is available for most of the countries from the year 2009.
- LTC beds (per 1000 65+ population): This indicator assesses the available care infrastructure in a country and measures the number of places in residential long-term care facilities for people aged 65+ in 2012. As it measures the availability of care provision, it is generally seen as a policy measure that supports defamilialization (through public provision) of care in a country. As for the carer support index, we use values from 2012 (except for Denmark, where only 2011 values were available).

**Figure 1.**
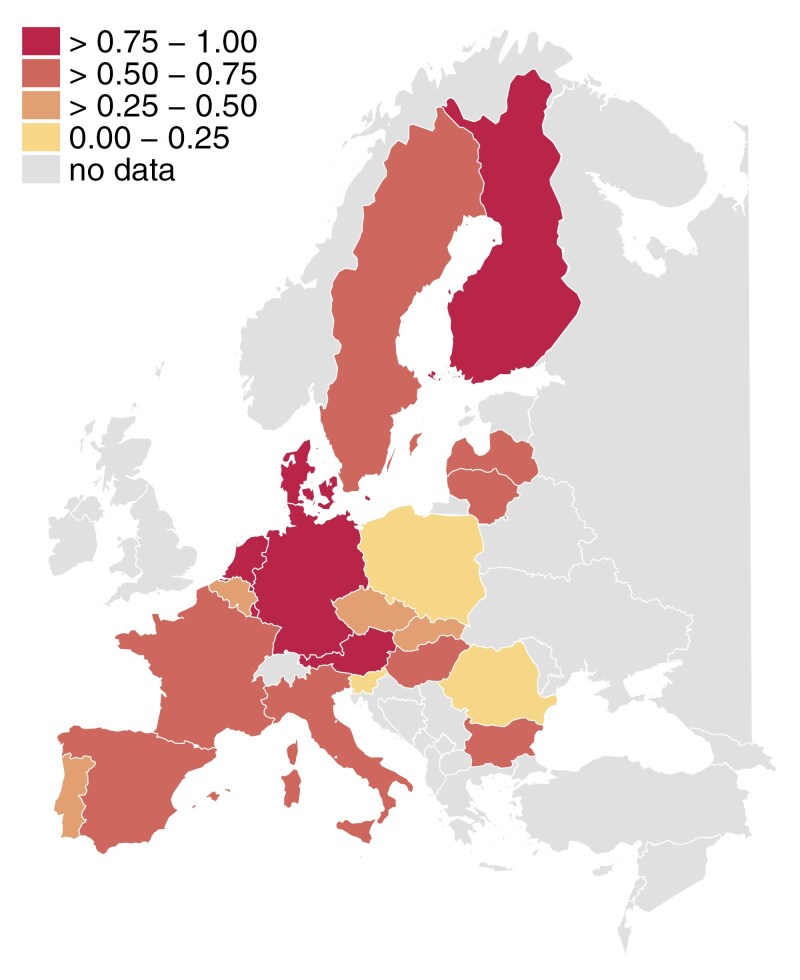
Caregiver support index across Europe.

Country details of the three policy indicators are summarized in Table 2, and Figure 1 presents a map with the distribution of the caregiver support index across Europe.

#### Additional variables

All additional variables were taken from baseline and are mainly included for propensity score matching. In addition to sex, country, and age (regrouped into four age groups), we considered respondents’ functional limitations, education, employment situation, number of children (not necessarily in the household), household size, partnership status, urbanicity, and the number of wave-participation together with information on first wave appearance. As a measure of functional limitations, we used an increased (two or more) number of limitations in performing instrumental activities of daily living (“IADL limitations”) based on six essential activities of an independent life. For the analyses, functional limitations were defined as having at least one IADL limitation. Education is measured according to the International Standard Classification of Educational Degrees (ISCED-97) and was regrouped into “low education” (preprimary, primary or lower secondary education), “medium education” (secondary or postsecondary education), and “high education” (first and second stages of tertiary education or higher). The employment situation measures whether respondents are in paid work or not. We count both biological and non-biological children for the number of children (continuous). The household size counts all people living in the household (irrespective of legal relationship), and we additionally include one binary variable assessing whether the partner was alive and lived in the household. Urbanicity is assessed with five categories, ranging from “a big city” (value 1) to 5 (“a rural area or village”). The number of waves the respondent participated in counts the number of waves in SHARE, and we also considered the wave at which the respondent first appeared in the survey (accounting for possible cohort effects). Details on each variable, including categories are shown in Table 1.

**Table 1.** Sample Description of Treatments, Raw Untreated, and Controls (Matched Untreated) for Men and Women

Variable	Categories or range	Women						Men					
		Treatments (carers) (*n* = 5,201)		Raw untreated (*n* = 29,354)		Matched untreated (*n* = 10,402)		Treatments (carers) (*n* = 3,847)		Raw untreated (*n* = 29,673)		Matched untreated (*n* = 7,694)	
		Obs. or mean	Col. % or (*SD*)	Obs. or mean	Col. % or (*SD*)	Obs. or mean	Col. % or (*SD*)	Obs. or mean	Col. % or (*SD*)	Obs. or mean	Col. % or (*SD*)	Obs. or mean	Col. % or (*SD*)
Age	50–59 years	1,939	37.3	14,142	48.2	4,039	38.8	1,214	31.6	11,897	40.1	2,448	31.8
	60–69 years	1,859	35.7	9,626	32.8	3,655	35.1	1,317	34.2	10,619	35.8	2,711	35.2
	70–79 years	1,170	22.5	4,556	15.5	2,239	21.5	1,017	26.4	5,779	19.5	1,992	25.9
	80–90 years	233	4.5	1,030	3.5	469	4.5	299	7.8	1,378	4.6	543	7.1
Wealth	High	1,481	28.5	9,157	31.2	3,036	29.2	1,111	28.9	9,804	33.0	2,216	28.8
	Medium	1,668	32.1	9,014	30.7	3,386	32.6	1,251	32.5	9,288	31.3	2,559	33.3
	Low	2,052	39.5	11,183	38.1	3,980	38.3	1,485	38.6	10,581	35.7	2,919	37.9
Education	High	854	16.4	6,190	21.1	1,639	15.8	796	20.7	7,273	24.5	1,559	20.3
	Medium	1,774	34.1	11,486	39.1	3,632	34.9	1,422	37.0	12,410	41.8	2,801	36.4
	Low	2,573	49.5	11,678	39.8	5,131	49.3	1,629	42.3	9,990	33.7	3,334	43.3
Functional limitations	Yes	977	18.8	3,930	13.4	1,851	17.8	482	12.5	2,472	8.3	951	12.4
	No	4,224	81.2	25,424	86.6	8,551	82.2	3,365	87.5	27,201	91.7	6,743	87.6
Employment situation	In paid work	1,215	23.4	10,218	34.8	2,478	23.8	1,100	28.6	11,753	39.6	2,192	28.5
	Not in paid work	3,986	76.6	19,136	65.2	7,924	76.2	2,747	71.4	17,920	60.4	5,502	71.5
Partner in household	Yes	4,835	93.0	26,618	90.7	9,671	93.0	3,726	96.9	28,833	97.2	7,451	96.8
	No	366	7.0	2,736	9.3	731	7.0	121	3.1	840	2.8	243	3.2
Urbanicity	A big city	758	14.6	4,415	15.0	1,516	14.6	497	12.9	4,279	14.4	949	12.3
	The suburbs or outskirts of a big city	610	11.7	3,344	11.4	1,222	11.7	468	12.2	3,398	11.5	924	12.0
	A large town	790	15.2	4,774	16.3	1,575	15.1	641	16.7	4,904	16.5	1,232	16.0
	A small town	1,328	25.5	6,957	23.7	2,711	26.1	1,057	27.5	6,981	23.5	2,223	28.9
	A rural area or village	1,715	33.0	9,864	33.6	3,378	32.5	1,184	30.8	10,111	34.1	2,366	30.8
Baseline wave	Wave 1	1,552	29.8	6,188	21.1	2,940	28.3	1,209	31.4	6,694	22.6	2,324	30.2
	Wave 2	748	14.4	2,765	9.4	1,467	14.1	565	14.7	2,865	9.7	1,096	14.2
	Wave 4	1,661	31.9	8,364	28.5	3,559	34.2	1,191	31.0	8,160	27.5	2,549	33.1
	Wave 5	716	13.8	5,426	18.5	1,440	13.8	551	14.3	5,512	18.6	1,108	14.4
	Wave 6	298	5.7	2,479	8.4	548	5.3	190	4.9	2,525	8.5	324	4.2
	Wave 7	146	2.8	2,552	8.7	304	2.9	89	2.3	2,277	7.7	183	2.4
	Wave 8	80	1.5	1,580	5.4	144	1.4	52	1.4	1,640	5.5	110	1.4
Household size	2–12	2.5	(0.9)	2.5	(0.9)	2.5	(0.9)	2.5	(1.0)	2.6	(1.0)	2.5	(1.0)
Number of children	0–17	2.4	(1.4)	2.3	(1.3)	2.4	(1.4)	2.3	(1.5)	2.3	(1.3)	2.3	(1.4)
Wave participations	2–9	5.2	(0.9)	4.1	(1.8)	5.1	(1.9)	5.1	(1.9)	4.1	(1.7)	5.1	(2.0)

*Note*s: Col. = Column; Obs. = Observations; SD = standard deviation.

### Analytical Strategy

This study compares the mental health trajectories of older men and women who became carers (i.e., the “treatments”) with trajectories of matched same-sex participants who remained non-carers (i.e., the “controls”). All analyses were conducted for men and women separately. To identify matched controls who did not become carers—but who were considered to have a similar probability for becoming carers (based on observed characteristics)—we applied propensity score matching (PSM) combined with exact matching (Green & Stuart, 2014). PSM is a widely used strategy to estimate treatment effects when randomized experimental designs are not feasible and to address confounding in observational studies (Rosenbaum & Rubin, 1983). Broadly speaking, PSM consists of two steps. The first step is to match observed treatments in the data (9,048 treatments in our case) with controls taken from our raw sample that have similar profiles (but remain non-carers). For this, propensity scores of becoming a carer are estimated with a regression model that uses treatment (i.e., transition into personal care) as a dependent variable and includes various covariates as predictors. With these individual scores, cases that have similar propensities are then identified as controls. After matching treatment and controls (and deliberately restricting the sample to treatment and matched controls), the second step is then to compare both groups (without additionally “adjusting” for covariates when estimating treatment effects). We used the “kmatch” procedure in Stata (Jann, 2017) and calculated propensity scores for men and women separately using logit models with all aforementioned additional variables (incl. wealth) as predictors—excluding country, which was used for exact matching (requiring that matching is conducted with controls from the same country only). As a matching algorithm, we applied nearest-neighbor matching (1:2, without replacement), which finds the two closest observations in a country. In sum, this resulted in a total matched sample of 27,144 respondents (9,048 treatments and 18,096 controls) that are used in subsequent analyses to compare trajectories. As a test of robustness, supplementary analyses were conducted with alternative matching strategies (i.e., nearest-neighbor matching with replacement and kernel matching), all leading to similar results. Table 1 gives—separately for men and women—a sample description for the treatment group, the matched controls (or “matched untreated”), and the raw untreated, thus, allowing to check the balance between treatment and controls.

To compare mental health trajectories between treatment and controls, we used longitudinal data on mental health from the same waves for both groups (Lacey et al., 2024). For example, in the case that a respondent (treatment case) participated in three waves (e.g., waves 4–6) and began caring at wave 5, we used wave 5 mental health scores of the matched controls for comparison. Equally, in this example, wave 4 data was used to compare scores one wave before transition, and wave 6 for one wave after the transition. Mental scores were centered at 0 for each respondent, allowing us to analyze trajectories in relation to when respondents became carers. In other words, for the comparisons, we did as if the identified controls began caring at the same wave as the treatment case and compared trajectories in relation to when becoming a carer. This allows us to compare mental health trajectories before (prior “−1”), during (between “−1” and “0”), and after the transition into care (after “0”) between treatments (with different waves when starting personal care) and their matched controls. With a maximum observation period of 9 waves, we could theoretically assess mental health up to 8 before and 7 waves after transition into care (when care started either at wave 9 or wave 2, respectively). However, this was rare and limited to countries that were already in SHARE at wave 1. Therefore, we focused on mental health scores available 4 waves before or after the transition—allowing each respondent to contribute as many observations as he or she provided that fell within this range (thereby not requiring 9 observations per respondent).

**Figure 2.**
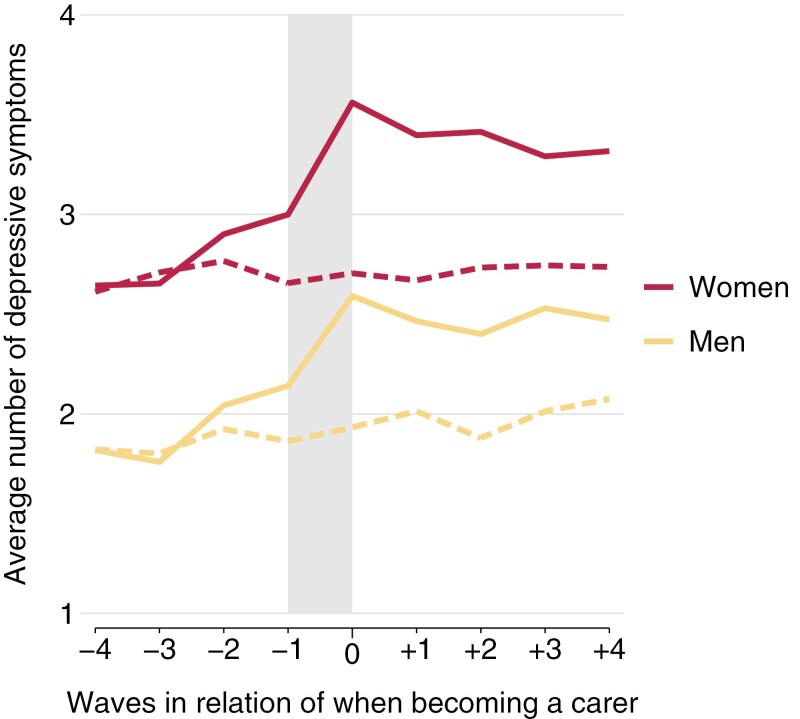
Mental health trajectories for men and women (treatment and controls). Shaded area indicates the transition period when becoming a carer. Solid lines indicate trajectories for carers (treatments), and dashed lines in the same color for respective matched non-carers (controls).

**Figure 3.**
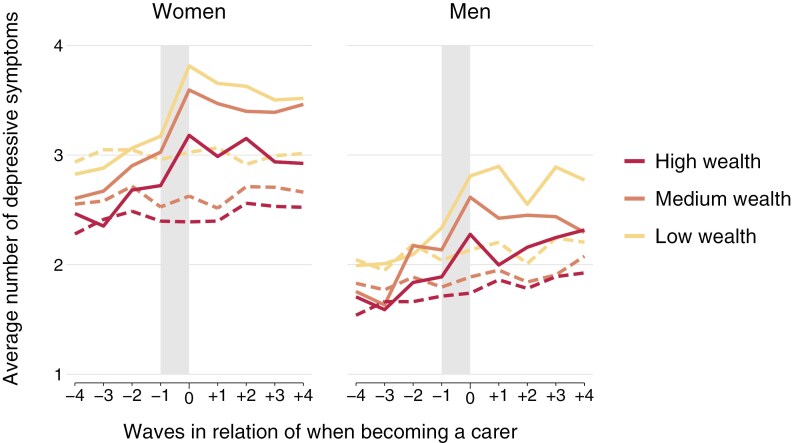
Mental health trajectories for men and women (treatment and controls) by levels of wealth. Shaded area indicates the transition period when becoming a carer. Solid lines indicate trajectories for carers (treatments), and dashed lines in the same color for respective matched non-carers (controls).

**Figure 4.**
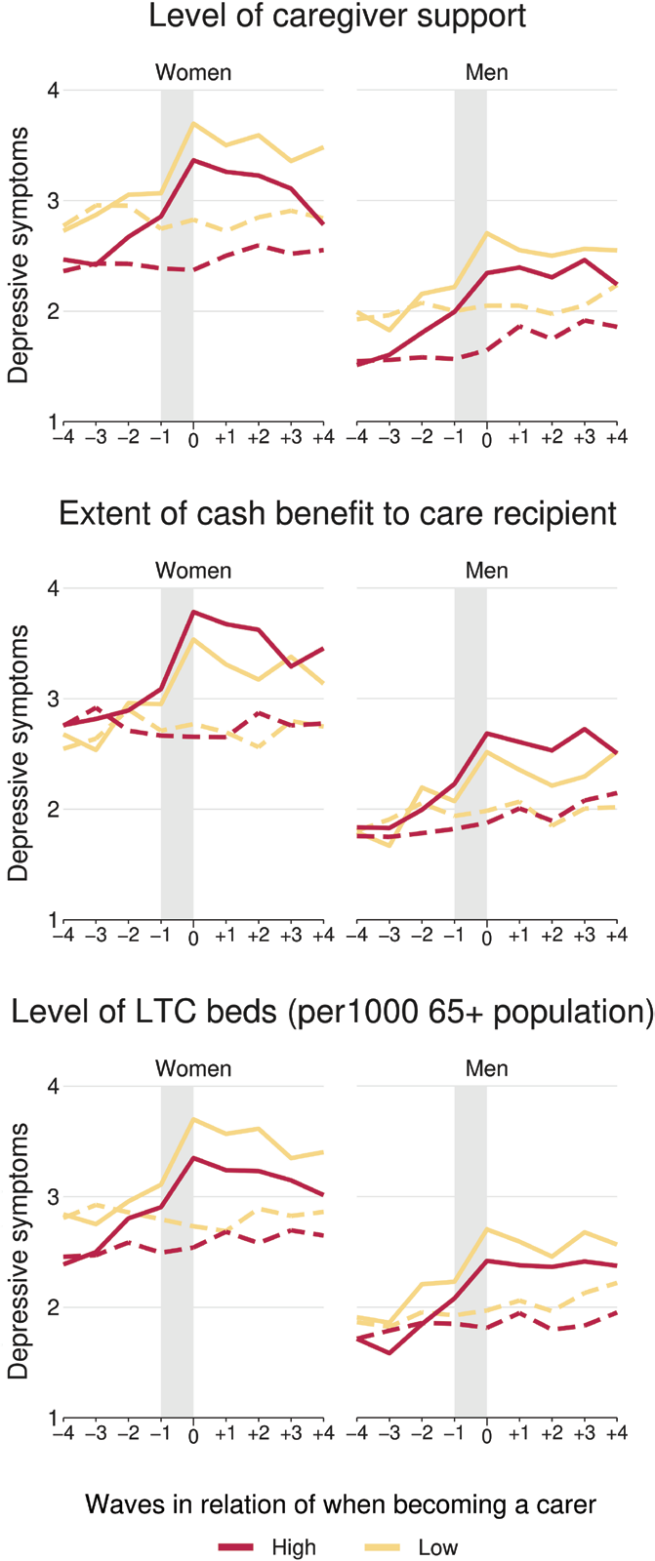
Mental health trajectories for men and women (treatment and controls) by LTC policies. Shaded area indicates the transition period when becoming a carer. Solid lines indicate trajectories for carers (treatments), and dashed lines in the same color for respective matched non-carers (controls). LTC = Long-term care.

The results are presented in Figure 2, showing trajectories for men and women, followed by Figures 3 and 4, which further distinguish by wealth or by levels of macro indicators. For the latter, countries were grouped into two groups based on their rank orders, where countries above the median were labeled “high” and those at or below as “low” (see Table 2 for details). Additionally, we estimated a series of piecewise growth curve models to predict mental health trajectories for treatments and controls, with treatment (becoming a carer) and waves in relation to when becoming a carer as main predictors. Hereby, the periods prior, during, and after becoming a carer were estimated as separate slopes in more detail based on spline models with two turning points (at year −1 and year 0). These models also considered the hierarchical structure of our data and accounted for the clustering of available observations within respondents and countries (i.e., three-level multilevel models with observations nested in respondents and countries). Results are presented in Supplementary Table S1, together with tests of interactions to compare the slopes between carers and non-carers (based on interactions between treatment and slopes). Then, Supplementary Table S2 additionally presents findings that compare slopes between wealth groups and macro indicators (and not between carers and non-carers) based on interaction terms between either wealth or macro indicators and slopes, again for men and women separately (shown in Supplementary Table S2). As our main interest is to investigate if the impact of personal care differs by wealth or policy (and not if trajectories of non-carers differ by these factors), Supplementary Table S2 presents differences by wealth and policy for carers only, whereas the figures included trajectories for all subgroups. All calculations and figures were produced with Stata (Version 18.0), and codes are available upon request for replication.

## Results

Table 1 compares men and women who became carers (treatment) with those who remained non-carers in the raw sample (raw untreated) or who were used as matched controls (matched untreated). Overall, compared with non-carers, carers tend to be older, have less formal education, have lower wealth, are less likely to be in paid employment, and are more likely to live in a rural area or village. As expected, we also see that becoming a carer is associated with a higher number of wave participations. When compared to the matched controls, for both men and women, we see that the treatment and control groups are well-balanced and that they are very similar along the just-mentioned characteristics (also used for the PSM).

Table 2 shows how LTC policy indicators vary by country. Looking at the index for caregiver support (also shown in Figure 1), carers receive more support in Scandinavian and Western European countries, while values are comparatively low in Eastern European countries. The second LTC indicator, levels of cash benefits for care recipients, is rather scattered around Europe, with the highest values in Austria, Germany, Spain, and Italy, as well as in the Czech Republic and Finland. Turning to the availability of LTC beds, we again see that Eastern countries have comparatively low values. Notably, none of the countries appeared to have a clear emphasis on one of the three types of measures, but countries with high levels of support for carers generally also had relatively high values for the remaining two measures.

Answers to the main research questions are presented in Figures 2–4 (mental health trajectories) together with Supplementary Tables S1 and S2 (estimates from spline models). Overall, three findings are worth noting: First, we observe clear differences in trajectories between carers and non-carers. Specifically, for carers, there is a comparatively higher increase of depressive symptoms in the waves before the transition period and a clearly more pronounced increase during the transition period (between wave −1 and wave 0). For female carers, for example, the transition period is accompanied by an increase in depressive symptoms of 0.54, and for matched non-carers the number of depressive symptoms is almost unchanged (−0.04). As presented in the third column of Supplementary Table S1, this corresponds to a difference between female carers and non-carers of 0.58 (95% CI: 0.50–0.67). Respective values for men are 0.43 for carers and 0.07 for non-carers, with a significant difference of 0.37 (0.28–0.46; see Supplementary Table S1 for more details). Second, the levels of depressive symptoms are generally higher for women and for lower wealth groups, but no clear differences exist by LTC policy indicators. In detail, at all stages of the trajectory, we see that the levels of depressive symptoms are generally higher for women compared with men (irrespective of caring status) and that higher wealth is also related to better mental health (i.e., lower number of depressive symptoms). In the cases of the macro indicators, however, the picture is less clear, with some support that countries with a high level of caregiver support or a high level of LTC beds have generally somewhat lower levels of depressive symptoms. Third, the increase of depressive symptoms for carers during the transition period slightly varies by gender and wealth, but no difference exists by LTC policy indicators. When comparing the changes in depressive symptoms during the transition period of male and female carers, for example, the increase appears slightly higher for women than men (0.54 for women and 0.43 for men), and we indeed also observe significant differences in additional analyses testing for an interaction based on a pooled sample of men and women (χ^2^ (1) = 6.7, *p* = .010, not shown in table). Likewise, we find support that the increase during the transition period is higher with lower wealth, particularly for women. Here, differences are 0.21 point higher (95% CI: 0.04–0.38) in case of low wealth compared with high wealth (see Supplementary Table S2 for details). For the macro indicators, though, results were rather unclear, and we could not find systematic differences for the three LTC policy indicators under study. The only exceptions exist for women, where the increase in depressive symptoms during the transition was slightly higher if countries had low levels of LTC beds and where the increase was slightly lower if cash benefits for care recipients were less generous.

## Discussion

Two main findings result from our analyses. First, starting to provide personal care on a regular basis within the household is associated with a substantial worsening of mental health and this worsening often begins before people become regular carers. This pattern of slight worsening before and marked worsening during the transition period holds true for both men and women and for different levels of wealth (albeit at different, overall higher levels for women and people with lower wealth). Second, the worsening of mental health during the transition period was slightly higher for women and for carers with lower wealth. However, we found no systematic differences according to the level of supportive LTC policy indicators, neither according to policies describing the extent to which carers are supported nor according to the extent to which care recipients are supported nor according to the public availability of care services. To our knowledge, this is the first study to compare mental health trajectories over an extended time period (up to four waves before and 4 years after transition to care) for older men and women across 28 European countries, and we also comprehensively explored—for the first time—potential differences in trajectories by three conceptually based types of national LTC policies (Verbakel et al., 2023).

The first finding is consistent with previous research, particularly studies showing that caring (especially intensive care within the household) is negatively associated with mental health, together with studies revealing similar associations based on alternative health outcomes, such as quality of life (Siegrist & Wahrendorf, 2009), self-perceived health (Kaschowitz & Brandt, 2017), or physical functioning (Lacey et al., 2024). By additionally showing that mental health often worsens before people start to provide care on a regular basis, we add new evidence that supports the idea that people often define or declare themselves rather late as carers (Lacey et al., 2024; Montgomery et al., 2007). On the other hand, though, this finding could also mean that respondents already provided personal care before, but not on a regular basis and this may have already affected their mental health. Or, our finding may reflect a decrease in health that is due to negative emotions when expecting to care regularly for someone in the future—sometimes also labeled as “anticipation effect” (Miceli & Castelfranchi, 2014). Similarly, participants may have had a close relative who became ill before they started caring for that person, and therefore, participants may have experienced distress (including feelings of sadness and worries) before they became actively involved in caring. However, as in previous studies, it remains an open question whether the health effects are related to the increased psychosocial and physical burden of caring itself (Pearlin et al., 1990), or to the inherent burdens and worries of having a relative in poor health matters (Hansen & Slagsvold, 2013; Litwin et al., 2014), or—and probably most likely—whether both aspects are mechanisms at play.

The second finding points to stronger mental health impacts for women and people in disadvantaged socioeconomic circumstances, but that carers’ trajectories are not affected by national policies. In case of women, this points to the fact that caring tasks are usually more demanding than for men (Eurocare, 2024), and thus, that women suffer more than men. Likewise, it seems plausible that disadvantaged population groups suffer more as they cannot afford assistance and therefore are possibly left alone with the task. Nevertheless, albeit the slope differences were statistically significant in our results, far-reaching conclusions remain difficult, and we need to keep in mind that an increase (albeit slightly steeper for women and low-wealth households) was clearly present in all cases. The fact that trajectories were not affected by national policies is a bit harder to explain. But a closer look into the literature seems helpful, suggesting that if studies focus on rather intense personal care within households, then, that the associations are similar across countries (Kaschowitz & Brandt, 2017), whereas variations are mostly found in studies that investigate less intensive care (Dujardin et al., 2011; Uccheddu et al., 2019; Verbakel, 2014). In other words, it seems that intensive personal care has an independent negative impact on health that cannot be attenuated by policies. This again leads to the aforementioned possible mechanisms for negative health impacts of care, and we may speculate that countries—in principle—can provide supportive LTC policies that attenuate health consequences to some extent, but less so if the care recipient is seriously ill. In that case, the inherent sadness, burdens, and worries of having a relative in poor health cannot be addressed through LTC policies. Importantly, however, this does not mean that LTC policies do not matter from a public health perspective. On the contrary, they surely do because they can reduce the extent to which people rely on family support (i.e., through defamilialization effects) and decrease the likelihood that someone starts to provide personal care (and therefore do affect population health). Our findings only suggest that, once a person is a carer and provides personal care within the household, then the policy measures under study may become less important. So LTC policies still have the potential to reduce the burden of caring in a country, as they reduce the number of intense burdensome care in a country. More detailed studies are required to draw far-reaching conclusions at this point, where the assessment of caring needs to be refined by including characteristics of the care situation.

This leads to several limitations of our study. First, and as just mentioned, while our study measured whether participants provided personal care within the household across waves of SHARE, we may ask whether patterns of mental health trajectories (and their variations between countries) would have been different if we had studied other types of care, for example, practical household help on a less regular basis. In fact, providing regular personal care within a household setting may involve more intensive caring responsibilities, leading to greater personal restrictions, increased worries, and more profound mental health effects than providing practical assistance in the household. Other aspects of the care situation may involve the location, care intensity (i.e., hours in a week), as well as information on the relationship of the carer to the care recipient (e.g., spouse or children with special needs), and details on his/her health conditions—or even information on whether the carer received professional support. In fact, all these aspects seem relevant for health-related consequences. However, albeit SHARE data provides some of these aspects, information is available for some waves only (e.g., for different types of care or relationship status in case of personal care), and thus, the investigation of mental health trajectories across an extended time period would have been limited. Furthermore, studying all these aspects would require an additional study with a different focus (and without country comparisons). Second, turning to the outcome under study, we could alternatively have used a cut-point to create a binary indicator for increased levels of depressive symptoms (to identify clinically relevant levels of symptoms). Yet, in additional analyses (not shown) findings were almost identical in case of a binary outcome. Likewise, it may also have been instructive to distinguish different subdimensions of depressive symptoms (e.g., distinguishing between somatic and affective symptoms). Third, albeit our policy measures were conceptually based and available for most SHARE countries, we still need to question their validity and reliability and avoid far-reaching conclusions about the role of LTC policies. For example, in some countries, LTC policies have changed more than in others, and therefore, the focus on 1 year may be problematic, as well as things may be very different after the COVID-19 pandemic. Furthermore, some indicators need to be criticized and interpreted with caution. For example, in case of available LTC beds in a country, the measure does not account for the general health of a population or age distribution, and therefore, fewer beds could also simply be due to a healthier or younger population (and not to a more generous policy) as well as it is not clear to what extent available LTC beds are publicly subsidized (and free for those in need of care; Verbakel et al., 2023). Furthermore, the availability of beds does not necessarily reflect its actual use, which is possibly more directly linked to the health of the caregiver. Some may argue that these measurement issues provide arguments to regroup countries into typologies (instead of using macro indicators of LTC as in our case; Bambra, 2007b; Esping-Andersen, 1990). We addressed this question in supplementary analyses (shown in Supplementary Figure S2) and again found that the trajectories were broadly similar across typologies (especially for men). One exception, however, was that the increase in depressive symptoms appeared somewhat more pronounced for women in Mediterranean countries than for women in other countries. This observation may indicate that caring tasks are particularly demanding for women in countries with more traditional gender orientations and thus suggests that cultural ideals of family care in a country, along with gender orientations, may be additional factors to consider in future analyses (see also Eggers et al., 2024; Floridi et al., 2022). Fourth, this study focused on the first transitions into care among respondents who were not caring at their first appearance in SHARE. As a result, prior caregiving episodes before study entry may have been missed. Likewise, a conclusion about the health effects of transitioning out of care is not possible based on our study, which would require a different sample of those caring at baseline who stopped caring (with additional PSM to identify controls) and—at the conceptual level—probably also involves different mechanisms (e.g., loneliness). Finally, although propensity score matching allowed us to consider a range of potential confounders, unobserved confounders remain an issue, as in all observational studies.

In conclusion, our study provides substantial support that starting personal in-home care on a regular basis is accompanied by mental health worsening, especially for women and carers in disadvantaged socioeconomic circumstances, and that health worsening often starts before the transition into personal care. Furthermore, we find that LTC policies did not affect the impact of caring on these trajectories—despite their importance in shaping the likelihood of caring. Our study also illustrates the importance of studying health trajectories over the course of an extended time period, and not to restrict analyses on changes of mental health before and after the transition into care only. These findings call for increased intervention efforts to improve the conditions of carers.

## Supplementary Material

gbaf053_suppl_Supplementary_Materials

## Data Availability

This paper uses data from SHARE Waves 1, 2, 3, 4, 5, 6, 7, 8 and 9 (DOIs: 10.6103/SHARE.w1.900, 10.6103/SHARE.w2.900, 10.6103/SHARE.w3.900, 10.6103/SHARE.w4.900, 10.6103/SHARE.w5.900, 10.6103/SHARE.w6.900, 10.6103/SHARE.w7.900, 10.6103/SHARE.w8.900, 10.6103/SHARE.w8ca.900, 10.6103/SHARE.w9.900, 10.6103/SHARE.w9ca900) see Börsch-Supan et al. (2013) for methodological details.

## References

[CIT0001] Bambra, C. (2007a). Defamilisation and welfare state regimes: A cluster analysis. International Journal of Social Welfare, 16(4), 326–338. https://doi.org/10.1111/j.1468-2397.2007.00486.x

[CIT0002] Bambra, C. (2007b). Going beyond the three worlds of welfare capitalism: Regime theory and public health research. Journal of Epidemiology and Community Health, 61(12), 1098–1102. https://doi.org/10.1136/jech.2007.064295 18000134 PMC2465657

[CIT0003] Borsch-Supan, A., Brandt, M., Hunkler, C., Kneip, T., Korbmacher, J., Malter, F., Schaan, B., Stuck, S., Zuber, S., & Team, S. C. C. (2013). Data resource profile: The survey of health, ageing and retirement in Europe (SHARE). International Journal of Epidemiology, 42(4), 992–1001. https://doi.org/10.1093/ije/dyt088 23778574 PMC3780997

[CIT0004] Courtin, E., Jemiai, N., & Mossialos, E. (2014). Mapping support policies for informal carers across the European Union. Health Policy, 118(1), 84–94. https://doi.org/10.1016/j.healthpol.2014.07.013 25132460

[CIT0005] Dujardin, C., Farfan-Portet, M. -I., Mitchell, R., Popham, F., Thomas, I., & Lorant, V. (2011). Does country influence the health burden of informal care? An international comparison between Belgium and Great Britain. Social Science & Medicine (1982), 73(8), 1123–1132. https://doi.org/10.1016/j.socscimed.2011.07.016 21855193

[CIT0006] Dunkle, R. E., Feld, S., Lehning, A. J., Kim, H., Shen, H. -W., & Kim, M. H. (2014). Does becoming an ADL spousal caregiver increase the caregiver’s depressive symptoms? Research on Aging, 36(6), 655–682. https://doi.org/10.1177/0164027513516152 25651543

[CIT0007] Eggers, T., Grages, C., & Pfau-Effinger, B. (2024). Gender and policies on paid family care: Overview of debate and theoretical reflections. Journal of Family Research, 36, 43–57. https://doi.org/10.20377/jfr-938

[CIT0008] Esping-Andersen, G. (1990). The three worlds of welfare capitalism. Polity Press.

[CIT0009] Eurocare. (2024). Later-life caring in Europe. Policy Report.

[CIT0010] Floridi, G., Quashie, N. T., Glaser, K., & Brandt, M. (2022). Partner care arrangements and well-being in mid- and later life: The role of gender across care contexts. Journals of Gerontology, Series B: Psychological Sciences and Social Sciences, 77(2), 435–445. https://doi.org/10.1093/geronb/gbab209 34752616 PMC8824554

[CIT0011] Green, K. M., & Stuart, E. A. (2014). Examining moderation analyses in propensity score methods: Application to depression and substance use. Journal of Consulting and Clinical Psychology, 82(5), 773–783. https://doi.org/10.1037/a0036515 24731233 PMC4172552

[CIT0012] Haidt, J., & Rodin, J. (1999). Control and efficacy as interdisciplinary bridges. Review of General Psychology, 3, 317–337. https://doi.org/10.1037//1089-2680.3.4.317

[CIT0013] Hajek, A., & König, H. H. (2016). The effect of intra- and intergenerational caregiving on subjective well-beingevidence of a population based longitudinal study among older adults in Germany. PLoS One, 11(2), e0148916. https://doi.org/10.1371/journal.pone.0148916 26859511 PMC4747461

[CIT0014] Hansen, T., & Slagsvold, B. (2013). The psychological effects of providing personal care to a partner: A multidimensional perspective. Health Psychology Research, 1(2), e25. https://doi.org/10.4081/hpr.2013.e25 26973910 PMC4768584

[CIT0015] Jann, B. (2017). KMATCH: Stata module for multivariate-distance and propensity-score matching.

[CIT0016] Kaschowitz, J., & Brandt, M. (2017). Health effects of informal caregiving across Europe: A longitudinal approach. Social Science & Medicine (1982), 173, 72–80. https://doi.org/10.1016/j.socscimed.2016.11.036 27930918

[CIT0017] Lacey, R. E., Xue, B. W., Di Gessa, G., Lu, W. T., & McMunn, A. (2024). Mental and physical health changes around transitions into unpaid caregiving in the UK: A longitudinal, propensity score analysis. Lancet Public Health, 9(1), e16–e25. https://doi.org/10.1016/S2468-2667(23)00206-2 37977176

[CIT0018] Lacey, R. E., Xue, B., & McMunn, A. (2022). The mental and physical health of young carers: A systematic review. Lancet Public Health, 7(9), e787–e796. https://doi.org/10.1016/S2468-2667(22)00161-X 36057277

[CIT0019] Leitner, S. (2003). Varieties of familialism - The caring function of the family in comparative perspective. European Societies, 5(4), 353–375. https://doi.org/10.1080/1461669032000127642

[CIT0020] Litwin, H., Stoeckel, K. J., & Roll, A. (2014). Relationship status and depressive symptoms among older co-resident caregivers. Aging & Mental Health, 18(2), 225–231. https://doi.org/10.1080/13607863.2013.837148 24047262

[CIT0021] McMunn, A., Bird, L., Webb, E., & Sacker, A. (2020). Gender divisions of paid and unpaid work in contemporary UK couples. Work, Employment and Society, 34(2), 155–173. https://doi.org/10.1177/0950017019862153

[CIT0022] McMunn, A., Nazroo, J., Wahrendorf, M., Breeze, E., & Zaninotto, P. (2009). Participation in socially-productive activities, reciprocity and wellbeing in later life: Baseline results in England. Ageing and Society, 29, 765–782. https://doi.org/10.1017/s0144686x08008350

[CIT0023] Miceli, M., & Castelfranchi, C. (2014). Expectancy and emotion. OUP Oxford.

[CIT0024] Montgomery, R. J., Rowe, J. M., & Kosloski, K. (2007). Family caregiving. In J. A. Blackburn & C. N. Dulmus (Eds.), Handbook of gerontology: Evidence‐based approaches to theory, practice, and policy (pp. 426–454). John Wiley & Sons.

[CIT0025] Pearlin, L. I., Mullan, J. T., Semple, S. J., & Skaff, M. M. (1990). Caregiving and the stress process—an overview of concepts and their measures. Gerontologist, 30(5), 583–594. https://doi.org/10.1093/geront/30.5.583 2276631

[CIT0026] Pinquart, M., & Sörensen, S. (2003). Differences between caregivers and noncaregivers in psychological health and physical health: A meta-analysis. Psychology and Aging, 18(2), 250–267. https://doi.org/10.1037/0882-7974.18.2.250 12825775

[CIT0027] Pinquart, M., & Sorensen, S. (2006). Gender differences in caregiver stressors, social resources, and health: An updated meta-analysis. Journals of Gerontology, Series B: Psychological Sciences and Social Sciences, 61(1), P33–45. https://doi.org/10.1093/geronb/61.1.p33 16399940

[CIT0028] Quashie, N. T., Wagner, M., Verbakel, E., & Deindl, C. (2022). Socioeconomic differences in informal caregiving in Europe. European Journal of Ageing, 19(3), 621–632. https://doi.org/10.1007/s10433-021-00666-y 36052200 PMC9424460

[CIT0029] Rosenbaum, P. R., & Rubin, D. B. (1983). The central role of the propensity score in observational studies for causal effects. Biometrika, 70(1), 41–55. https://doi.org/10.2307/2335942

[CIT0030] Siegrist, J., & Wahrendorf, M. (2009). Participation in socially productive activities and quality of life in early old age: Findings from SHARE. Journal of European Social Policy, 19(4), 317–326. https://doi.org/10.1177/1350506809341513

[CIT0031] Tur-Sinai, A., Teti, A., Rommel, A., Hlebec, V., & Lamura, G. (2020). How many older informal caregivers are there in Europe? Comparison of estimates of their prevalence from three European surveys. International Journal of Environmental Research and Public Health, 17(24), 9531. https://doi.org/10.3390/ijerph17249531 33352669 PMC7767284

[CIT0032] Uccheddu, D., Gauthier, A. H., Steverink, N., & Emery, T. (2019). The pains and reliefs of the transitions into and out of spousal caregiving. A cross-national comparison of the health consequences of caregiving by gender. Social Science & Medicine, 240, 112517. https://doi.org/10.1016/j.socscimed.2019.112517 31561110

[CIT0033] UNECE. (2019). The challenging roles of informal carers (Vol. 22). United Nations Economic Commission for Europe.

[CIT0034] Verbakel, E. (2014). Informal caregiving and well-being in Europe: What can ease the negative consequences for caregivers? Journal of European Social Policy, 24(5), 424–441. https://doi.org/10.1177/0958928714543902

[CIT0035] Verbakel, E. (2018). How to understand informal caregiving patterns in Europe? The role of formal long-term care provisions and family care norms. Scandinavian Journal of Public Health, 46(4), 436–447. https://doi.org/10.1177/1403494817726197 28823224 PMC5989248

[CIT0036] Verbakel, E., Glaser, K., Amzour, Y., Brandt, M., & Van Groenou, M. B. (2023). Indicators of familialism and defamilialization in long-term care: A theoretical overview and introduction of macro-level indicators. Journal of European Social Policy, 33(1), 34–51. https://doi.org/10.1177/09589287221115669

[CIT0037] Wahrendorf, M., Ribet, C., Zins, M., & Siegrist, J. (2008). Social productivity and depressive symptoms in early old age-results from the GAZEL study. Aging & Mental Health, 12(3), 310–316. https://doi.org/10.1080/13607860802120805 18728943 PMC2802287

