# Supplementary material for: Mental Health Trajectories of Men and Women Who Start Providing Personal Care: European Findings From SHARE Using Propensity Score Matching
Source: J Gerontol B Psychol Sci Soc Sci. 2025 Mar 13;80(6):gbaf053. doi: 10.1093/geronb/gbaf053 (PMC12084829; doi:10.1093/geronb/gbaf053)
Supplement: gbaf053_suppl_Supplementary_Materials [file gbaf053_suppl_supplementary_materials.docx]

**Supplementary material**


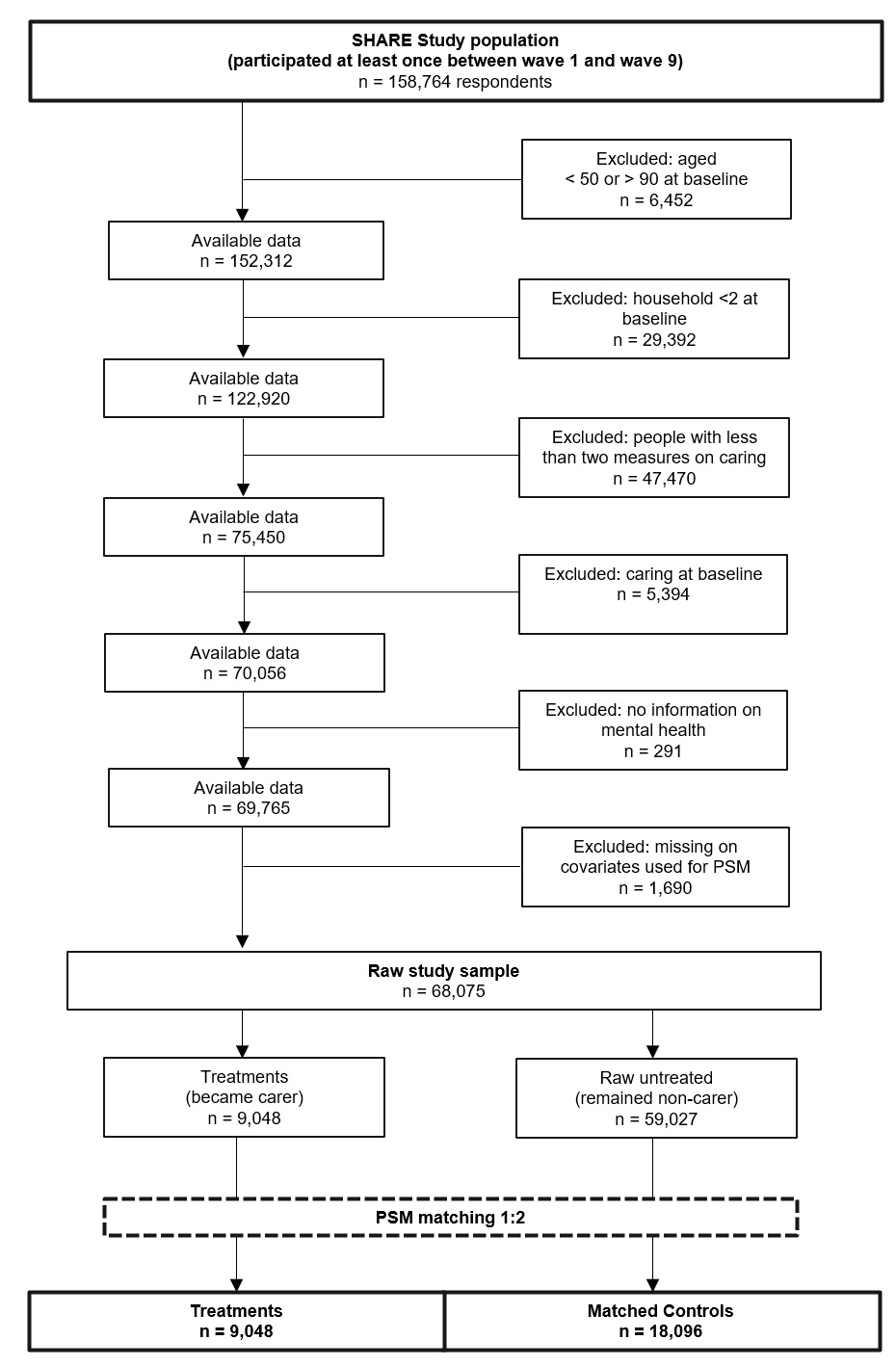


Supplementary Figure 1**:** Flow Chart

Supplementary Figure 2*:* Mental health trajectories for men and women (treatment and controls) by welfare state typology

Note. Shaded area indicates the transition period when becoming a carer. Solid lines indicate trajectories for carers (treatments), and dashed lines in the same colour for respective matched non-carers (controls). Countries are regrouped based on Esping-Andersen’s framework and subsequent modifications as follows: Mediterranean (Greece, Italy, Spain, Portugal, Malta, Cyprus), Post-Socialist (Bulgaria, Croatia, Czech Republic, Estonia, Hungary, Latvia, Lithuania, Poland, Romania, Slovakia, Slovenia), Conservative (Austria, Belgium, France, Germany, Luxembourg, Switzerland), and Social-democratic (Denmark, Finland, Sweden, Norway, Netherland)

Supplementary Table 1: Results of Spline Models for men and women including slope differences between carers and non-carers

|  |  | **Women** | | | | | | | |  | **Men** | | | | | | | |
| --- | --- | --- | --- | --- | --- | --- | --- | --- | --- | --- | --- | --- | --- | --- | --- | --- | --- | --- |
|  |  | Treatment (carers) | |  | Control (non-carers) | |  | Difference between carers  and non-carers | |  | Treatment (carers) | |  | Control (non-carers) | |  | Difference between carers  and non-carers | |
|  |  | Coef. | 95% CI |  | Coef. | 95% CI |  | Coef. | 95% CI |  | Coef. | 95% CI |  | Coef. | 95% CI |  | Coef. | 95% CI |
|  |  |  |  |  |  |  |  |  |  |  |  |  |  |  |  |  |  |  |
| **Total** |  |  |  |  |  |  |  |  |  |  |  |  |  |  |  |  |  |  |
| Before becoming a carer |  | 0.126 | (0.090/0.162) |  | 0.035 | (0.010/0.061) |  | 0.091 | (0.047/0.134) |  | 0.118 | (0.082/0.153) |  | 0.037 | (0.012/0.062) |  | 0.080 | (0.037/0.123) |
| Transition period |  | 0.542 | (0.473/0.610) |  | -0.044 | (-0.096/0.009) |  | 0.585 | (0.499/0.671) |  | 0.434 | (0.363/0.505) |  | 0.067 | (0.011/0.122) |  | 0.367 | (0.277/0.458) |
| After becoming a carer |  | -0.052 | (-0.080/-0.024) |  | 0.056 | (0.036/0.075) |  | -0.108 | (-0.142/-0.074) |  | 0.031 | (-0.001/0.062) |  | 0.076 | (0.055/0.098) |  | -0.046 | (-0.084/-0.007) |
|  |  |  |  |  |  |  |  |  |  |  |  |  |  |  |  |  |  |  |
| **High wealth** |  |  |  |  |  |  |  |  |  |  |  |  |  |  |  |  |  |  |
| Before becoming a carer |  | 0.123 | (0.057/0.189) |  | 0.040 | (-0.007/0.087) |  | 0.083 | (0.002/0.164) |  | 0.074 | (0.009/0.139) |  | 0.055 | (0.009/0.100) |  | 0.019 | (-0.060/0.098) |
| Transition period |  | 0.416 | (0.288/0.543) |  | -0.069 | (-0.166/0.028) |  | 0.485 | (0.324/0.645) |  | 0.357 | (0.225/0.488) |  | 0.024 | (-0.078/0.126) |  | 0.333 | (0.167/0.499) |
| After becoming a carer |  | -0.042 | (-0.093/0.009) |  | 0.069 | (0.034/0.103) |  | -0.111 | (-0.173/-0.049) |  | 0.012 | (-0.045/0.068) |  | 0.076 | (0.037/0.115) |  | -0.064 | (-0.133/0.004) |
| **Medium Wealth** |  |  |  |  |  |  |  |  |  |  |  |  |  |  |  |  |  |  |
| Before becoming a carer |  | 0.126 | (0.064/0.189) |  | 0.026 | (-0.018/0.069) |  | 0.101 | (0.025/0.177) |  | 0.144 | (0.085/0.203) |  | 0.023 | (-0.019/0.066) |  | 0.121 | (0.048/0.193) |
| Transition period |  | 0.554 | (0.432/0.676) |  | -0.036 | (-0.128/0.055) |  | 0.591 | (0.438/0.743) |  | 0.428 | (0.305/0.551) |  | 0.065 | (-0.031/0.161) |  | 0.362 | (0.206/0.519) |
| After becoming a carer |  | -0.044 | (-0.093/0.006) |  | 0.055 | (0.022/0.089) |  | -0.099 | (-0.159/-0.039) |  | -0.004 | (-0.058/0.050) |  | 0.080 | (0.043/0.117) |  | -0.084 | (-0.149/-0.019) |
| **Low wealth** |  |  |  |  |  |  |  |  |  |  |  |  |  |  |  |  |  |  |
| Before becoming a carer |  | 0.127 | (0.069/0.185) |  | 0.040 | (-0.002/0.083) |  | 0.087 | (0.016/0.158) |  | 0.124 | (0.064/0.183) |  | 0.035 | (-0.007/0.077) |  | 0.089 | (0.016/0.161) |
| Transition period |  | 0.624 | (0.515/0.732) |  | -0.024 | (-0.110/0.062) |  | 0.648 | (0.509/0.786) |  | 0.500 | (0.384/0.616) |  | 0.104 | (0.012/0.197) |  | 0.396 | (0.248/0.544) |
| After becoming a carer |  | -0.065 | (-0.111/-0.019) |  | 0.050 | (0.017/0.084) |  | -0.115 | (-0.172/-0.059) |  | 0.089 | (0.036/0.142) |  | 0.075 | (0.038/0.112) |  | 0.014 | (-0.051/0.078) |
|  |  |  |  |  |  |  |  |  |  |  |  |  |  |  |  |  |  |  |
| **Level of caregiver support high** |  |  |  |  |  |  |  |  |  |  |  |  |  |  |  |  |  |  |
| Before becoming a carer |  | 0.121 | (0.062/0.180) |  | 0.037 | (-0.005/0.079) |  | 0.084 | (0.012/0.156) |  | 0.152 | (0.096/0.207) |  | 0.022 | (-0.018/0.062) |  | 0.130 | (0.061/0.198) |
| Transition period |  | 0.593 | (0.476/0.710) |  | -0.009 | (-0.100/0.083) |  | 0.602 | (0.453/0.750) |  | 0.391 | (0.275/0.507) |  | 0.134 | (0.042/0.227) |  | 0.256 | (0.108/0.405) |
| After becoming a carer |  | -0.057 | (-0.107/-0.008) |  | 0.084 | (0.050/0.118) |  | -0.141 | (-0.201/-0.081) |  | 0.074 | (0.022/0.127) |  | 0.108 | (0.072/0.144) |  | -0.034 | (-0.097/0.030) |
| **Level of caregiver support low** |  |  |  |  |  |  |  |  |  |  |  |  |  |  |  |  |  |  |
| Before becoming a carer |  | 0.149 | (0.096/0.201) |  | 0.020 | (-0.016/0.057) |  | 0.128 | (0.064/0.192) |  | 0.117 | (0.066/0.169) |  | 0.054 | (0.018/0.090) |  | 0.063 | (0.000/0.126) |
| Transition period |  | 0.526 | (0.428/0.625) |  | -0.048 | (-0.123/0.028) |  | 0.574 | (0.450/0.698) |  | 0.459 | (0.357/0.561) |  | 0.009 | (-0.071/0.089) |  | 0.450 | (0.320/0.580) |
| After becoming a carer |  | -0.063 | (-0.104/-0.023) |  | 0.040 | (0.011/0.068) |  | -0.103 | (-0.152/-0.054) |  | 0.001 | (-0.044/0.046) |  | 0.061 | (0.030/0.092) |  | -0.060 | (-0.114/-0.005) |
|  |  |  |  |  |  |  |  |  |  |  |  |  |  |  |  |  |  |  |
| **Cash benefit to care recipient high** |  |  |  |  |  |  |  |  |  |  |  |  |  |  |  |  |  |  |
| Before becoming a carer |  | 0.154 | (0.097/0.210) |  | 0.023 | (-0.018/0.063) |  | 0.131 | (0.062/0.201) |  | 0.166 | (0.110/0.222) |  | 0.057 | (0.018/0.097) |  | 0.109 | (0.040/0.177) |
| Transition period |  | 0.718 | (0.609/0.827) |  | -0.071 | (-0.156/0.014) |  | 0.789 | (0.650/0.927) |  | 0.479 | (0.368/0.589) |  | 0.097 | (0.010/0.185) |  | 0.381 | (0.240/0.522) |
| After becoming a carer |  | -0.068 | (-0.113/-0.024) |  | 0.069 | (0.037/0.100) |  | -0.137 | (-0.192/-0.083) |  | 0.037 | (-0.012/0.086) |  | 0.097 | (0.064/0.131) |  | -0.060 | (-0.120/-0.001) |
| **Cash benefit to care recipient low** |  |  |  |  |  |  |  |  |  |  |  |  |  |  |  |  |  |  |
| Before becoming a carer |  | 0.086 | (0.022/0.149) |  | 0.060 | (0.015/0.105) |  | 0.026 | (-0.052/0.104) |  | 0.111 | (0.051/0.170) |  | 0.038 | (-0.004/0.081) |  | 0.072 | (-0.001/0.145) |
| Transition period |  | 0.500 | (0.382/0.618) |  | -0.078 | (-0.167/0.012) |  | 0.577 | (0.429/0.725) |  | 0.334 | (0.214/0.455) |  | 0.007 | (-0.086/0.101) |  | 0.327 | (0.174/0.480) |
| After becoming a carer |  | -0.076 | (-0.124/-0.028) |  | 0.047 | (0.014/0.080) |  | -0.123 | (-0.181/-0.065) |  | 0.022 | (-0.033/0.077) |  | 0.070 | (0.033/0.107) |  | -0.048 | (-0.114/0.018) |
|  |  |  |  |  |  |  |  |  |  |  |  |  |  |  |  |  |  |  |
| **Level of LTC beds high** |  |  |  |  |  |  |  |  |  |  |  |  |  |  |  |  |  |  |
| Before becoming a carer |  | 0.154 | (0.096/0.212) |  | 0.021 | (-0.020/0.062) |  | 0.132 | (0.061/0.203) |  | 0.121 | (0.066/0.176) |  | 0.052 | (0.013/0.091) |  | 0.069 | (0.002/0.136) |
| Transition period |  | 0.435 | (0.321/0.548) |  | 0.075 | (-0.014/0.163) |  | 0.360 | (0.216/0.504) |  | 0.404 | (0.290/0.518) |  | 0.018 | (-0.074/0.110) |  | 0.386 | (0.239/0.532) |
| After becoming a carer |  | -0.033 | (-0.081/0.014) |  | 0.056 | (0.023/0.088) |  | -0.089 | (-0.146/-0.031) |  | 0.052 | (0.002/0.102) |  | 0.066 | (0.031/0.100) |  | -0.014 | (-0.075/0.048) |
| **Level of LTC beds low** |  |  |  |  |  |  |  |  |  |  |  |  |  |  |  |  |  |  |
| Before becoming a carer |  | 0.123 | (0.074/0.172) |  | 0.036 | (0.001/0.072) |  | 0.087 | (0.026/0.147) |  | 0.134 | (0.084/0.183) |  | 0.041 | (0.006/0.077) |  | 0.092 | (0.031/0.153) |
| Transition period |  | 0.604 | (0.512/0.697) |  | -0.136 | (-0.206/-0.065) |  | 0.740 | (0.624/0.856) |  | 0.431 | (0.333/0.528) |  | 0.052 | (-0.024/0.127) |  | 0.379 | (0.256/0.502) |
| After becoming a carer |  | -0.072 | (-0.110/-0.035) |  | 0.068 | (0.041/0.094) |  | -0.140 | (-0.186/-0.094) |  | 0.022 | (-0.021/0.065) |  | 0.098 | (0.068/0.127) |  | -0.076 | (-0.128/-0.023) |
|  |  |  |  |  |  |  |  |  |  |  |  |  |  |  |  |  |  |  |

Supplementary Table 2: Results of Spline Models including slope differences for carers between wealth groups and levels of policy indicator (for carers only)

|  |  | **Women** | | | | |  | **Men** | | | | |
| --- | --- | --- | --- | --- | --- | --- | --- | --- | --- | --- | --- | --- |
|  |  | Treatments (carers) | |  | Difference within group  (vs. high) | |  | Treatments (carers) | |  | Difference within group  (vs. high) | |
|  |  | Coef. | 95% CI |  | Coef. | 95% CI |  | Coef. | 95% CI |  | Coef. | 95% CI |
|  |  |  |  |  |  |  |  |  |  |  |  |  |
| **High wealth** |  |  |  |  |  |  |  |  |  |  |  |  |
| Before becoming a carer |  | 0.123 | (0.057/0.189) |  | - |  |  | 0.074 | (0.009/0.139) |  | - |  |
| Transition period |  | 0.416 | (0.288/0.543) |  | - |  |  | 0.357 | (0.225/0.488) |  | - |  |
| After becoming a carer |  | -0.042 | (-0.093/0.009) |  | - |  |  | 0.012 | (-0.045/0.068) |  | - |  |
| **Medium Wealth** |  |  |  |  |  |  |  |  |  |  |  |  |
| Before becoming a carer |  | 0.126 | (0.064/0.189) |  | 0.003 | (-0.087/0.094) |  | 0.144 | (0.085/0.203) |  | 0.070 | (-0.018/0.158) |
| Transition period |  | 0.554 | (0.432/0.676) |  | 0.139 | (-0.038/0.316) |  | 0.428 | (0.305/0.551) |  | 0.071 | (-0.109/0.251) |
| After becoming a carer |  | -0.044 | (-0.093/0.006) |  | -0.001 | (-0.073/0.070) |  | -0.004 | (-0.058/0.050) |  | -0.016 | (-0.094/0.063) |
| **Low wealth** |  |  |  |  |  |  |  |  |  |  |  |  |
| Before becoming a carer |  | 0.127 | (0.069/0.185) |  | 0.004 | (-0.083/0.092) |  | 0.124 | (0.064/0.183) |  | 0.050 | (-0.038/0.138) |
| Transition period |  | 0.624 | (0.515/0.732) |  | 0.208 | (0.040/0.376) |  | 0.500 | (0.384/0.616) |  | 0.143 | (-0.032/0.318) |
| After becoming a carer |  | -0.065 | (-0.111/-0.019) |  | -0.023 | (-0.091/0.046) |  | 0.089 | (0.036/0.142) |  | 0.077 | (-0.001/0.155) |
|  |  |  |  |  |  |  |  |  |  |  |  |  |
| **Level of caregiver support high** |  |  |  |  |  |  |  |  |  |  |  |  |
| Before becoming a carer |  | 0.121 | (0.062/0.180) |  | - |  |  | 0.152 | (0.096/0.207) |  | - |  |
| Transition period |  | 0.593 | (0.476/0.710) |  | - |  |  | 0.391 | (0.275/0.507) |  | - |  |
| After becoming a carer |  | -.057 | (-0.107/-0.008) |  | - |  |  | 0.074 | (0.022/0.127) |  | - |  |
| **Level of caregiver support low** |  |  |  |  |  |  |  |  |  |  |  |  |
| Before becoming a carer |  | 0.149 | (0.096/0.201) |  | 0.028 | (-0.051/0.107) |  | 0.117 | (0.066/0.169) |  | -0.035 | (-0.110/0.041) |
| Transition period |  | 0.526 | (0.428/0.625) |  | -0.067 | (-0.219/0.086) |  | 0.459 | (0.357/0.561) |  | 0.068 | (-0.086/0.223) |
| After becoming a carer |  | -0.063 | (-0.104/-0.023) |  | -0.006 | (-0.070/0.058) |  | 0.001 | (-0.044/0.046) |  | -0.073 | (-0.142/-0.004) |
|  |  |  |  |  |  |  |  |  |  |  |  |  |
| **Cash benefit to care recipient high** |  |  |  |  |  |  |  |  |  |  |  |  |
| Before becoming a carer |  | 0.154 | (0.097/0.210) |  | - |  |  | 0.166 | (0.110/0.222) |  | - |  |
| Transition period |  | 0.718 | (0.609/0.827) |  | - |  |  | 0.479 | (0.368/0.589) |  | - |  |
| After becoming a carer |  | -0.068 | (-0.113/-0.024) |  | - |  |  | 0.037 | (-0.012/0.086) |  | - |  |
| **Cash benefit to care recipient low** |  |  |  |  |  |  |  |  |  |  |  |  |
| Before becoming a carer |  | 0.086 | (0.022/0.149) |  | -0.068 | (-0.153/0.017) |  | 0.111 | (0.051/0.170) |  | -0.055 | (-0.137/0.027) |
| Transition period |  | 0.500 | (0.382/0.618) |  | -0.218 | (-0.379/-0.058) |  | 0.334 | (0.214/0.455) |  | -0.144 | (-0.308/0.020) |
| After becoming a carer |  | -0.076 | (-0.124/-0.028) |  | -0.008 | (-0.074/0.057) |  | 0.022 | (-0.033/0.077) |  | -0.015 | (-0.089/0.058) |
|  |  |  |  |  |  |  |  |  |  |  |  |  |
| **Level of LTC beds high** |  |  |  |  |  |  |  |  |  |  |  |  |
| Before becoming a carer |  | 0.154 | (0.096/0.212) |  | - |  |  | 0.121 | (0.066/0.176) |  | - |  |
| Transition period |  | 0.435 | (0.321/0.548) |  | - |  |  | 0.404 | (0.290/0.518) |  | - |  |
| After becoming a carer |  | -0.033 | (-0.081/0.014) |  | - |  |  | 0.052 | (0.002/0.102) |  | - |  |
| **Level of LTC beds low** |  |  |  |  |  |  |  |  |  |  |  |  |
| Before becoming a carer |  | 0.123 | (0.074/0.172) |  | -0.031 | (-0.107/0.045) |  | 0.134 | (0.084/0.183) |  | 0.013 | (-0.061/0.087) |
| Transition period |  | 0.604 | (0.512/0.697) |  | 0.170 | (0.024/0.316) |  | 0.431 | (0.333/0.528) |  | 0.027 | (-0.123/0.177) |
| After becoming a carer |  | -0.072 | (-0.110/-0.035) |  | -0.039 | (-0.100/0.021) |  | 0.022 | (-0.021/0.065) |  | -0.030 | (-0.096/0.036) |
|  |  |  |  |  |  |  |  |  |  |  |  |  |
